# A Three-Armed Randomized Controlled Trial to Evaluate the Effectiveness, Acceptance, and Negative Effects of *StudiCare Mindfulness,* an Internet- and Mobile-Based Intervention for College Students with No and “On Demand” Guidance

**DOI:** 10.3390/ijerph20043208

**Published:** 2023-02-11

**Authors:** Ann-Marie Küchler, Dana Schultchen, Tim Dretzler, Morten Moshagen, David D. Ebert, Harald Baumeister

**Affiliations:** 1 Department of Clinical Psychology and Psychotherapy, Ulm University, 89081 Ulm, Germany; 2 Department of Clinical and Health Psychology, Ulm University, 89081 Ulm, Germany; 3 Department of Quantitative Methods in Psychology, Ulm University, 89081 Ulm, Germany; 4 Department for Sport and Health Sciences, Technical University of Munich, 80992 Munich, Germany

**Keywords:** college students, adherence, guidance, mindfulness, stress, depression, internet- and mobile-based interventions, e-Health, effectiveness, negative effects

## Abstract

The college years can be accompanied by mental distress. Internet- and mobile-based interventions (IMIs) have the potential to improve mental health but adherence is problematic. Psychological guidance might promote adherence but is resource intensive. In this three-armed randomized controlled trial, “guidance on demand” (GoD) and unguided (UG) adherence-promoting versions of the seven-module IMI StudiCare Mindfulness were compared with a waitlist control group and each other. The GoD participants could ask for guidance as needed. A total of 387 students with moderate/low mindfulness were recruited. Follow-up assessments took place after 1 (t1), 2 (t2), and 6 (t3) months. Post-intervention (t2), both versions significantly improved the primary outcome of mindfulness (*d* = 0.91–1.06, 95% CI 0.66–1.32) and most other mental health outcomes (*d* = 0.25–0.69, 95% CI 0.00–0.94) compared with WL, with effects generally persisting after 6 months. Exploratory comparisons between UG and GoD were mostly non-significant. Adherence was low but significantly higher in GoD (39%) vs. UG (28%) at the 6-month follow-up. Across versions, 15% of participants experienced negative effects, which were mostly mild. Both versions effectively promoted mental health in college students. Overall, GoD was not associated with substantial gains in effectiveness or adherence compared with UG. Future studies should investigate persuasive design to improve adherence.

## 1. Introduction

For many students, college is one of the most exciting times of their lives. However, this period can also be very stressful, as students are faced with various challenges such as settling into a new living situation, building a new social network, and establishing an academic routine [1]. A total of 75% of college students report moderate to high stress levels [2] and one-third screen positive for lifetime DSM-IV (Diagnostic and Statistical Manual of Mental Disorders, 4th ed. [3]) mental disorders—such as anxiety (24%) and mood disorders (21%) [4]—which are associated with impaired academic functioning and college dropout [5]. However, external and internal barriers such as a busy college schedule, the preference to self-manage problems, or the fear of stigmatization can prevent affected students from seeking professional support [6]. Consequently, two-thirds of students do not seek or receive adequate help [7].

A promising approach to overcome barriers to help seeking could be internet- and mobile-based interventions (IMIs), which can be accessed whenever and wherever desired and can also be used anonymously. Guided IMIs additionally incorporate trained professionals (such as psychologists) to support participants throughout the intervention (e.g., via motivation, feedback). These interventions have demonstrated equivalent effectiveness to face-to-face interventions across many application areas of physical and mental health. Additionally, they have the benefit of being potentially scalable [8]. For these reasons, IMIs seem well-suited for mental health promotion among college students [9]. This is supported by previous findings that suggest IMIs might reach students that would not otherwise seek help [10] and by existing evidence of IMI effectiveness in college student populations [11].

Mindfulness-based interventions—such as Mindfulness-based Stress Reduction (MBSR) [12] and Acceptance Commitment Therapy (ACT) [13]—could constitute a beneficial theoretical foundation for IMIs aiming to promote mental health in college students. The transdiagnostic, resource-oriented nature of these programs helps to make them low-stigma and broadly applicable. Mindfulness-based interventions teach that responding to external and internal events (such as thoughts, feelings, and bodily sensations) in an open, accepting, and non-judgmental manner fosters psychological well-being and resiliency to stress [14]. ACT extends this concept via the principle of “committed action”, which refers to being aware of and striving towards one’s own goals and values [15]. A 97-trial meta-analysis investigated the effectiveness of mindfulness-based IMIs (compared with different types of control groups) and found significant increases in mindfulness and decreases in depression, anxiety, and stress (*g* = 0.26–0.44, 95% CI 0.18 to 0.55) in both clinical and non-clinical samples [16,17]. Twenty-seven of the included trials examined college student samples, which underlines the effectiveness of mindfulness-based IMIs in this specific target group. Additionally, another meta-analysis (51 trials) examined the effectiveness of mindfulness-based interventions in college students [18], including 5 RCTs on mindfulness-based IMIs. Four of these RCTs identified significant effects on various mental health outcomes—such as mindfulness, stress, or self-compassion—compared with passive control groups. However, these studies share methodological limitations such as small sample sizes. Finally, a randomized controlled trial conducted by our research group evaluated the effectiveness of the guided IMI StudiCare Mindfulness (StudiCare-M) [19] in a sample of *N* = 149 college students. The intervention was found to effectively enhance mindfulness (*d* = 1.37, 95% CI 1.01 to 1.73) and mental quality of life (*d* = 0.59, 95% CI 0.26 to 0.91) and to reduce depression (*d* = −0.87, 95% CI −1.20 to −0.53), anxiety (*d* = −0.50, 95% CI −0.82 to −0.17), and stress (*d* = −0.92, 95% CI −1.25 to −0.58) compared with a waitlist control group.

This high effectiveness of StudiCare-M might partly be rooted in the fact that the intervention participants were guided by trained psychologists, or “e-coaches”. Evidence suggests that guided IMIs are superior to unguided ones both in terms of effectiveness (*standardized mean difference* (*SMD)* = −0.27, 95% CI: −0.45 to −0.10) and adherence (*SMD* = 0.52, 95% CI 0.37 to 0.67), with the increase in effectiveness potentially resulting from the increased adherence [20]. However, such therapeutic guidance is accompanied by an increased need for therapeutic resources and increased costs, which could impede large-scale dissemination [20,21]. An unguided version of StudiCare-M could be a low-cost alternative but might be associated with reduced adherence and effectiveness. Two potential solutions could help alleviate this dilemma. Firstly, unguided interventions could be adapted to include persuasive design elements in order to optimize adherence and effectiveness. These include general elements (e.g., a variety of delivery formats such as audio and video, automated reminders, goal-setting techniques, tracking/diaries) and mindfulness-specific elements (e.g., self-reflection and meditation exercises) [22,23]. Alternatively, interventions could employ the concept of “guidance on demand” (GoD). This is a potentially cost- and resource-reducing, tailored form of minimal guidance, wherein participants request e-coach support only when they actually need it, thereby preventing any unnecessary allocation of costly e-coach time [20]. Only a few studies have examined GoD-IMIs and their comparative effectiveness and adherence rates in relation to other guidance formats. Two studies that investigated cognitive behavioral IMIs (ICBT) for social phobia [24] and tinnitus [25] did not find any significant differences in GoD compared with unguided IMI versions. Another trial [26] compared fully guided ICBT for somatic symptom distress with a GoD version of the same IMI and found no significant differences between the two. These are interesting results, considering the general scientific consensus regarding the supposed superiority of guided IMIs [20]. A potential explanation might be that other factors—such as the precise operationalization of GoD, the nature of the sample, or the intervention approach—may influence the relationship between guidance, adherence, and effectiveness. More research is needed to gain insights into these questions. Concerning the specific field of mindfulness-based IMIs, evidence is even scarcer. While some study findings suggest that persuasive design elements can have a positive impact on the adherence and effectiveness of mindfulness-based IMIs [23], to the best of our knowledge, no previous study has examined the implementation of GoD or compared different guidance formats in the context of these interventions. 

The investigation of negative effects of psychological interventions has also been neglected so far, both in general as well as in the specific fields of IMIs [27] and mindfulness-based interventions [28]. Evidence of face-to-face mindfulness-based interventions suggests that meditation-related adverse effects occur in about 40% of participants, which is similar to the rate of new and worsening symptoms caused by psychotherapy [29]. To the best of our knowledge, no trial so far has examined the negative effects of a mindfulness-based IMI. However, examining negative effects of IMIs is important from both an ethical and legal standpoint and this delivery mode might bring about specific negative effects worth investigating, such as dropout due to technical issues [27].

Informed by the considerations listed here, the present study evaluated the effectiveness and adherence of a revised version of the previously developed IMI StudiCare-M (which has now been adapted to better promote adherence) in an unguided and a GoD version. It was hypothesized that participants of both versions would display significantly improved primary (mindfulness) and secondary (various mental health metrics, such as depression, anxiety, stress, well-being) outcomes compared with a waitlist control group 1, 2, and 6 months after randomization. Additionally, we examined whether there are any differences between the unguided and GoD version of StudiCare-M concerning effectiveness, adherence, satisfaction, and negative intervention effects.

## 2. Methods 

### 2.1. Study Design

**Figure 1:**
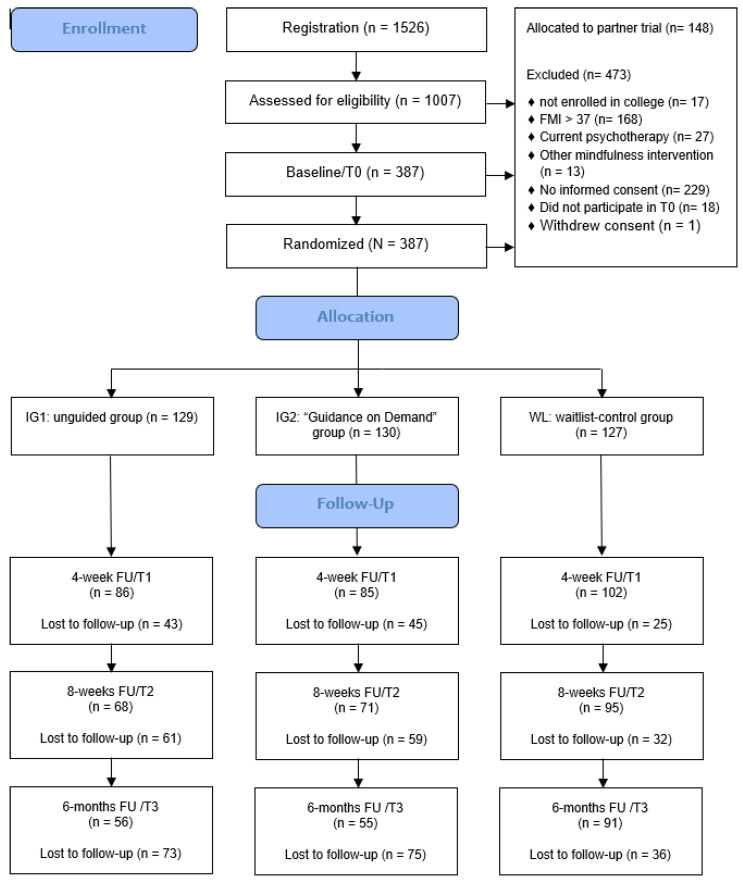
Flowchart.

In this three-armed, randomized controlled trial (RCT), we evaluated the effectiveness of two versions of the internet- and mobile-based intervention (IMI) StudiCare Mindfulness (StudiCare-M) compared against a waitlist control group (WL). Intervention group 1 (UG) received the unguided version of the intervention, whereas intervention group 2 (GoD) additionally received “guidance on demand” (see flowchart, Figure 1). Both versions were also exploratorily compared with each other to inform on potential differences in effectiveness, adherence, satisfaction, and negative effects. Participants of all three conditions had access to treatment as usual (TAU) and the use of other support options was monitored and controlled for. The trial was part of StudiCare (www.studicare.com, accessed on 7 February 2023), a project funded by BARMER health insurance that investigates and promotes college student mental health by providing a portfolio of IMIs to address various psychological and behavioral issues (e.g., resilience, physical activity, stress, procrastination, anxiety) [19,30,31,32]. StudiCare is the German branch of the “WHO World Mental Health Survey International College Student” project (WMH-ICS). This RCT was conducted and reported in concordance with the CONSORT 2010 statement [33]. It was registered a priori at the WHO International Clinical Trials Registry Platform via the German Clinical Studies Trial Register (TRN: DRKS00014774; registration date: 18 May 2018). In addition, a study protocol was published [34].

### 2.2. Eligibility Criteria

To be included in the trial, participants needed to provide written informed consent. Additionally, the following inclusion criteria had to be met: (a) age 18 or older, (b) enrolled in college or university, (c) sufficient knowledge of the German language, (d) internet access, (e) moderate to low mindfulness (Freiburg Mindfulness Inventory FMI < 37, corresponding to FMI mean in subjects from the general population) [35]. Exclusion criteria consisted of participation in psychotherapy or any kind of mindfulness intervention at the time of the screening.

### 2.3. Setting/Recruitment

Recruitment took place from May 2018 to November 2020. College students were recruited via flyers and posters, social media, student unions, and student counseling. However, the main recruitment channel was the dispatch of circular e-mails sent out each semester by over 15 cooperating colleges in Germany, Austria, and Switzerland. Students received information about StudiCare, including a link to the StudiCare homepage (www.studicare.com, accessed on 7 February 2023) where they could find detailed descriptions of the StudiCare IMIs and the opportunity to register. After passing a screening, students were allocated based on their college affiliation either to a partner trial (students from Ulm University and other Ulm colleges) or the present trial (all other colleges). The partner trial by Schultchen and colleagues [36] evaluated a fully guided version of StudiCare-M, incorporating psychobiological markers that required on-site assessment. In a next step, participants of the present trial had to provide written consent and complete baseline assessment. Following this, participants were randomized into one of three groups. Participants of both intervention groups immediately received access to the respective version of StudiCare-M. WL participants were provided with information on further study procedures and received access to the intervention after completion of the follow-up assessment (after 6 months).

### 2.4. Randomization

After baseline assessment, an independent researcher blinded to all study processes randomly allocated participants to the three study groups. An automated, online randomization program [37] was used to perform permuted block randomization with an allocation ratio of 1:1:1 and randomly arranged variable block sizes of 6, 9, and 12.

### 2.5. Intervention 

StudiCare-M is a refined and extended version of an IMI developed and evaluated in a previous RCT [19]. The revision included the addition of 2 core modules, resulting in 7 sequential core modules designed to be worked through on a weekly basis in 45–60 min each. Additionally, 2 booster modules were added (unlocked 4 and 12 weeks after module 7) to ensure intervention sustainability. This resulted in a recommended duration of about 8 weeks for the core intervention and 6 months for the entire intervention including the booster modules. However, participants’ access to the intervention was not limited to this timeframe and they were able to work through the intervention at their own pace. The modules provided information on stress, well-being, and mindfulness with a different focus each week (e.g., interoception, dysfunctional thinking, values and goals). For a detailed description of the topics and contents of each module, see Table 1. Besides a varied design—including texts, images, and many mandatory, interactive elements (e.g., self-reflection exercises)—StudiCare-M also contained weekly alternating mindfulness exercises such as body scans and breathing meditations. Participants received homework assignments between modules, in which they were encouraged to practice regularly with downloadable audio files and document their practice in a mindfulness diary. The content of the intervention was based on elements of Acceptance and Commitment Therapy (ACT) [13], Mindfulness-Based Stress Reduction (MBSR) [12], and general stress management techniques [38]. Participants were able to access the intervention on the Minddistrict platform (www.minddistrict.com, accessed 7 February 2023) via their personal username and password on a 24/7 basis. All transferred data were secured based on ISO27001 and the guidelines of NEN7510.

#### 2.5.1. Guidance and Promotion of Adherence

Participants assigned to the GoD version of the intervention were given access to written, asynchronous e-coach support through the Minddistrict platform’s messaging function. They could request feedback on their entries after completing a module or ask any questions as needed. E-coaches, who were psychologists trained and supervised by the authors (H.B. and A.K.), would respond to requests within two business days. They provided semi-standardized written feedback based on an e-coach manual, using predefined text modules that were slightly adapted to individual participants’ entries. Feedback typically consisted of one DIN A4 page per intervention module and the content focused on positive reinforcement, motivation, and encouragement. Additionally, e-coaches were available to answer any other questions the participants had, e.g., about the module content or technical issues. In the first module, a welcoming message was sent to each participant by their e-coach including a short introduction on how to use GoD. In contrast, participants receiving the unguided version of StudiCare-M (UG) were not provided with e-coach guidance. Instead, they received short standardized automated feedback messages at the end of each module for reinforcement and motivation. Additionally, participants of both intervention groups were sent automated standardized e-mails by the Minddistrict platform if they had not logged in for more than a week. Finally, participants of both intervention groups were given the opportunity to sign up for an SMS coach that sent out standardized automated text messages every 2 days for 8 weeks. Messages reminded participants of their homework assignments and motivated and prompted them to integrate mindfulness into their everyday life (e.g., “Pay attention to your body signals today: What do you notice when running to the bus? Taking a hot shower? Eating your dinner?”; “The journey is the destination—implementing a more mindful daily routine isn’t always easy. Don’t get discouraged and keep at it. It’s worth it!”). 

#### 2.5.2. Control Condition 

As in the IGs, WL participants had unrestricted access to usual treatment options (TAU). Additionally, an information leaflet about alternative support options (e.g., university counselling services, psychotherapy, and helplines) was provided and participants were encouraged to seek help in case of deterioration of well-being. Six months after randomization (t3), WL participants received the unguided version of the StudiCare-M.

### 2.6. Assessments and Outcomes

The assessment took place before (t0; baseline) and 4 weeks (t1; intermediate), 8 weeks (t2; post-intervention), and 6 months (t3; follow-up) after randomization (see Figure 1). At the intermediate assessment (t1) only a subset of outcomes was assessed. The online survey platform “Unipark” (www.unipark.com, accessed on 7 February 2023) was used for data collection. 

#### 2.6.1. Primary Outcome 

Mindfulness was assessed as the primary outcome at 8 weeks (t2; post-intervention) with the 14-item short scale of the Freiburg Mindfulness Inventory (FMI) [35] on a 4-point scale ranging from 1 = “rarely” to 4 = “almost always”. In previous research, it has demonstrated high internal consistency (α = 0.84) [39] and sensitivity to change [35].

#### 2.6.2. Secondary Outcomes 

*Mindfulness at intermediate assessment (t1) and follow-up (t3).* The FMI was additionally assessed after 4 weeks and 6 months. 

*Depressive symptoms.* Within the depression module of the Patient Health Questionnaire (PHQ-9) [40], 9 items are rated on a 4-point scale (0 = “not at all” to 3 = “nearly every day”). The PHQ-9 was shown to have good diagnostic properties and a high internal consistency of α = 0.89 [41].

*Anxiety.* As a screening instrument for anxiety, the 7-item Generalized Anxiety Disorder Questionnaire [42] was used. Its 7 items range from “not at all” (=0) to “nearly every day” (=3). It has excellent internal consistency (α = 0.89) [43].

*Stress.* To assess participants’ perceived level of stress, the 4-item short form of the Perceived Stress Scale [44] (PSS-4) was used. The items of this scale range from 0 = “never” to 4 = “very often”. Internal consistency was shown to be Cronbach’s α = 0.77 [45].

*Well-Being.* Subjective psychological well-being was measured with the 5-item World Health Organization Well-Being Index [46]. The scale ranges from “at no time” (=0) to “all of the time” (=5) and its clinical validity has been demonstrated to be very high [47].

*Academic outcomes.* With a modified version [30] of the Presenteeism Scale for Students (SPS) [48] presenteeism, loss of productivity, and absenteeism were assessed. Presenteeism was measured by the subscale for work impairment (Work Impairment Scale; 10 items, scale range 1–5, transformed range 20–100), productivity losses by an adapted version of the Presenteeism Scale for Students’ work output scale, and absenteeism by inquiring on hours of absenteeism. Sufficient test–retest reliability and validity were previously shown for the Work Impairment Scale [48].

*Interoceptive sensibility.* To assess interoceptive sensibility (IS), we chose the awareness section of the Body Perception Questionnaire (BPQ) [49], which comprises 26 items of subjective identifications of bodily signals on a 5-point scale, ranging from “never” (=1) to “always” (=5). High scores reflect poor IS. The BPQ has good internal reliability with categorical omega coefficients between 0.77 and 0.96 [50].

*Self-efficacy.* The 10-item Self-efficacy Scale (SES) [51] was used to measure perceived general self-efficacy on a 4-point scale ranging from “1 = not at all true” to “4 = very true”. In previous research, the SES has demonstrated good internal consistency of 0.75–0.91 [52].

*Cognitive fusion.* To measure cognitive fusion, the Cognitive Fusion Questionnaire (CFQ-D) [53] was used. The seven items were rated on a 7-point scale ranging from “1 = never true” to “7 = always true”. Internal consistency has been found to be high with Cronbach’s α = 0.95 [53].

*Emotion regulation.* Individual differences in the habitual use of two emotion regulation strategies (cognitive reappraisal, 6 items; expressive suppression, 4 items) were assessed using the Emotion Regulation Questionnaire (ERQ) [54]. Agreement with each statement was indicated on a 7-point scale ranging from 1 (=strongly disagree) to 7 (=strongly agree). The ERQ has previously demonstrated good internal consistency (reappraisal: α = 0.74; suppression: α = 0.76) [55]. 

*Alexithymia.* The Toronto Alexithymia Scale (TAS-20) [56,57] contains 20 items rated on a 5-point scale (1 = strongly disagree; 5 = strongly agree). It has three factor scales: “difficulty identifying feelings” (DIF), “difficulty describing feelings” (DDF), and “externally oriented thinking” (EOF). The TAS-20 was found to have good internal consistency (Cronbach’s α = 0.85–0.86) [58].

*Negative Effects (only IGs).* A 22-item version of the Inventory for the Assessment of Negative Effects of Psychotherapy (INEP) [59] adapted specifically for IMIs was used. A total of 17 items rated on a 7-point bipolar scale (−3 = “worse”, +3 = “better”) assessed changes experienced during or after the intervention in different areas of life (e.g., social, work) as well as whether these were attributed to StudiCare-M. Only negative effects attributed to the intervention were analyzed. The other 5 items measured potential negative experiences associated with content and e-coaching (e.g., hurtful statements) and were rated on a 4-point scale (0 = “no agreement”, 3 = “full agreement”). The original scale was shown to have high internal consistency (α = 0.86) [59].

*Intervention satisfaction and adherence (only IGs).* An adapted version of the Client Satisfaction Questionnaire (CSQ-8) to specifically evaluate IMIs was chosen to assess intervention satisfaction [60]. Each of the 8 items was measured on a 4-point scale of specific response alternatives (e.g., 1 = “quite unsatisfied”, 4 = “very satisfied”). A Cronbach’s α between 0.88 and 0.92 has been previously demonstrated [61]. Intervention adherence was operationalized by the number of completed modules. Thereby, “per protocol” adherence was defined as the percentage of participants completing at least 5 of the 7 core modules 8 weeks after randomization (t2). Because participants were able to work through the intervention at their own pace and access to the intervention was not limited to 8 weeks (t2), follow-up adherence (6 months after randomization) was recorded at t3. Quantitative and qualitative data on participants’ satisfaction with various aspects of the intervention (e.g., number and length of modules, SMS coach, and practicability in daily life) were additionally collected using self-constructed items (e.g., “Which elements did you find particularly helpful?”). We also recorded the number of times participants of the GoD condition contacted their e-coach and whether participants of both UG and GoD subscribed to the SMS coach. Weekly time spent practicing mindfulness exercises was assessed retrospectively (at t2) via participant self-report.

#### 2.6.3. Covariates

Various sociodemographic as well as other variables were assessed: age, gender, nationality, marital status, study course and number of semesters, previous experience with mindfulness (assessed retrospectively at t2), psychotherapy experience, and the use of additional treatment options. Additionally, the Credibility Expectancy Questionnaire (CEQ) was assessed [62]. A total of 6 items—three for the credibility sub-scale (“how believable, convincing, and logical the treatment is” [63]) and three for the expectancy sub-scale (“improvements that clients believe will be achieved” [63])—were measured on a 9-point Likert scale. Higher scores indicated positive expectations and credibility of StudiCare-M. Internal consistency was found previously to be high, with Cronbach’s α = 0.84–0.85.

### 2.7. Statistical Analyses

Statistical analyses were conducted using IBM SPSS (version 28) [64] and R (version 4.0.3)) [65] with a significance level of α = 0.05 (two-sided). 

A priori sample size calculation (see study protocol by Küchler et al. [34] for details) based on a power of 1-ß = 0.9, α = 0.05, *intraclass correlation coefficient* (*ICC)* = 0.02, and an effect size of *d* = 0.40 [21] resulted in a sample size of *n* = 129 participants per group (*N* = 387).

Analyses were performed on an intention-to-treat basis, employing multiple imputations by chained equations [66] and assuming data to be missing at random [67]. *N* = 20 data sets were imputed with predictive mean matching [68]. We conducted all ITT analyses for each imputed data set and then pooled results according to Rubin’s rule [69]. Additionally, per-protocol analyses were calculated to analyze the potential influence of intervention adherence on effectiveness. According to our definition of adherence, these sub-analyses (UG: *n* = 26; GoD: *n* = 36) included all UG and GoD participants that had completed at least 5 of the 7 core modules at t2.

General linear models (GLM) were employed to explore group differences between UG and WL, GoD and WL (both primary analyses), and between UG and GoD (secondary analysis) 4 weeks (t1), 8 weeks (t2), and 6 months (t3) after randomization. For each group comparison, outcome variable, and assessment time, a linear regression model was applied, corrected for baseline values of the respective outcome variable. Dichotomous variables were dummy coded and continuous variables were *z*-standardized. Means (*M*), standard deviations (*SD*), standardized regression coefficients (*β*), and 95% confidence intervals (CI) were presented. Additionally, Cohen’s *d* (between group) and 95% CIs were calculated. According to Cohen’s rule of thumb [70], *d* = 0.2 was interpreted as a small, *d* = 0.5 as a medium, and *d* = 0.8 as a large effect.

To calculate the number of participants achieving reliable improvement in mindfulness (FMI) from pre- (t0) to post-intervention (t2), participants were coded as responders and non-responders according to the Reliable Change Index (RCI) [71]. Negative effects on the individual level were also determined using the RCI by calculating the number of participants that displayed a reliable deterioration from t0 to t2. Both reliable improvement and reliable deterioration were calculated based on based on α = 0.84 [39]. Chi-square tests were conducted to test for group differences in categorical variables (percentage of participants experiencing reliable improvement/deterioration, adherence, and negative effects) and t-tests were conducted for continuous variables (satisfaction). 

To examine the influence of guidance- and adherence-associated variables on the dependent variables “number of modules completed at t2” and “FMI at t2”, additional exploratory regression analyses were conducted using complete case data of the two IGs together. For “number of modules completed at t2”, we explored the predictor “SMS Coach signup” for both IGs and “number of e-coach contacts” for GoD. For “FMI at t2”, we examined the predictors “SMS Coach signup”, “number of modules completed at t2”, “average days of mindfulness practice per week”, and “average minutes per mindfulness practice day” for both IGs together, and additionally “number of e-coach contacts” for GoD. First, univariate associations between potential predictors and the respective dependent variable were investigated. Afterwards, a final multivariate model was established including all significant predictors. 

## 3. Results

### 3.1. Participants

In total, 1526 students registered for the trial. Of 1007 students taking part in the eligibility screening, 225 were excluded for various reasons (see Figure 1 for details), mostly because their initial FMI score was greater than 37 (*n* = 168). Of the remaining 782 participants, all 148 Ulm University students were allocated to the partner trial [36]. A total of 229 participants did not provide informed consent, 18 did not participate in the baseline assessment, and 1 withdrew consent during trial (waitlist control group, WL). This resulted in *N* = 386 participants. In addition, one participant from the WL was accidentally handled as if allocated to GoD during the trial. Consequently, for the purpose of data analysis they were relocated to the GoD group. This resulted in *n* = 129 (UG), *n* = 130 (GoD) and *n* = 127 (WL). In total, 113 participants (attrition rate: 29.3%) were lost at the 4-week follow-up (UG: 33.3%, GoD: 34.6%, WL: 19.7%), 152 (attrition rate: 39.4%) at the 8-week follow-up (UG: 47.3%, GoD: 45.4%, WL: 25.2%), and 202 (attrition rate: 52.3%) at the 6-month follow-up (UG: 56.6%, GoD: 57.7%, WL: 28.3%) (see flowchart, Figure 1).

The distributions of baseline characteristics were comparable between conditions (see Table 2). The mean age of participants was 26 years (*M* = 25.77, *SD* = 5.34). A total of 3/4 were female (74.9%) and most were single (66.1%) and of German citizenship (80.3%). On average, participants were in their ninth semester of college (*M* = 9.05, *SD* = 5.39), were full-time students (82.1%), and were enrolled in a wide range of courses, most prominently in the field of medicine and health (18.4%). Concerning previous help seeking, 23% of participants had received previous psychotherapy and 38% had some kind of experience with mindfulness (mainly self-study, e.g., internet, books, or audio). On average, treatment credibility was moderate to high with *M* = 20.36 (*SD* = 3.78; range 0–27) and treatment expectancy was a little lower with *M* = 18.15 (*SD* = 4.04; range 0–27). Concerning alternative support options, *n =* 119 participants at t1 (30.8%) and *n* = 122 at t2 (31.5%) indicated that they utilized a mode of support outside StudiCare Mindfulness (StudiCare-M) to improve their mental well-being (ITT sample; *N* = 386). Of these, 7.9/10.1% (at t1 and t2, respectively) consulted a psychologist or psychotherapist and 3.1/3.1% consulted a doctor and/or took psychopharmaceuticals. A total of 19.8/18.3% indicated that they had used other support offers. These percentages were comparable between groups and assessments.

### 3.2. Primary Outcome Analyses

**Figure 2:**
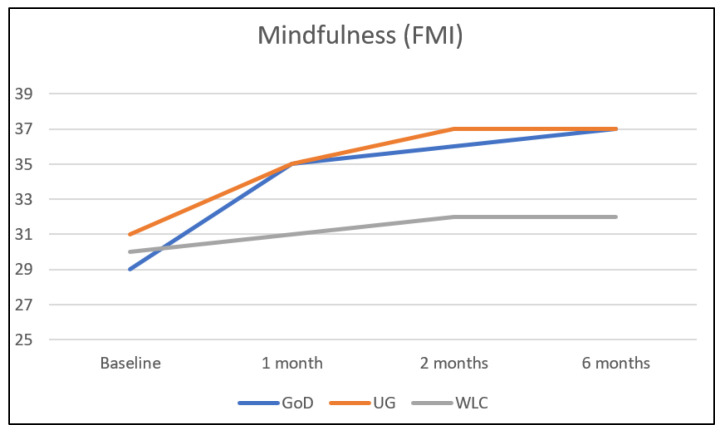
Primary outcome mindfulness in relation to study condition and assessment time.

A total of 8 weeks after randomization (t2), mindfulness was significantly improved by 0.88 standard deviations in both intervention groups compared with the waitlist control group (UG vs. WL: β = 0.88, 95% CI: 0.62 to 1.13, *p* > 0.001; GoD vs. WL: β = 0.88, 95% CI: 0.63 to 1.13, *p* > 0.001) (see Table 3 and Figure 2). Corresponding effect sizes were *d* = 1.06 (95% CI: 0.80; 1.32) for UG vs. WL and *d* = 0.91 (95% CI: 0.66; 1.17) for GoD vs. WL (see Table 4). Differences between beta and effect sizes are due to the fact that Cohen’s *d* does not take baseline differences into account. 

### 3.3. Secondary Outcome Analyses

Concerning t1 (4 weeks after randomization) and t3 (6 months after randomization), mindfulness was significantly improved in both intervention groups (IGs) compared with WL (see Table 3 and Figure 2). Significant improvement in both UG and GoD compared with WL could also be shown for most secondary outcome variables at most assessment times, as can be seen in Table 3. Exceptions were the academic outcomes (which could not be shown to be significantly improved at any assessment time in either UG or GoD compared with WL), interoceptive sensibility (which was only improved at one assessment time (t2) in one group—UG), and some other outcomes at some individual assessment times, mostly concerning UG (see Table 3). 

Comparisons between GoD and UG yielded mostly non-significant results (see Table 3), with the exceptions of anxiety at t3 (β = −0.28, 95% CI: −0.52 to −0.03, *p* = 0.026), stress at t2 (β = −0.29, 95% CI: −0.56 to −0.03, *p* = 0.031), and WHO-5 at t1 (β = 0.32, 95% CI: 0.05 to 0.60, *p* = 0.023), where improvement was found to be significantly higher in GoD compared with UG. 

**Table 3:** Results of regression analyses (intention-to-treat).

	*M* ± *SD*			Primary Analyses				Secondary Analysis	
				UG vs. WL		GoD vs. WL		UG vs. GoD	
Variable	UG (*n* = 129)	GoD (*n* = 130)	WL (*n* = 127)	β (95% CI)	*p*	β (95% CI)	*p*	β (95% CI)	*p*
Primary Outcome									
Mindfulness (FMI)									
Baseline 4 weeks 8 weeks 6 months	30.56 ± 4.74 34.59 ± 4.75 36.84 ± 4.86 36.49 ± 5.28	29.29 ± 4.91 34.45 ± 4.53 36.19 ± 5.10 37.26 ± 5.12	29.71 ± 4.71 30.75 ± 5.32 31.50 ± 5.17 31.89 ± 5.29	0.65 [0.41; 0.88] 0.88 [0.62; 1.13] 0.73 [0.49; 0.97]	**<0.001** **<0.001** **<0.001**	0.76 [0.53; 0.99] 0.88 [0.63; 1.13] 0.97 [0.69; 1.25]	**<0.001** **<0.001** **<0.001**	0.07 [−0.17; 0.31] −0.02 [−0.30; 0.26] 0.22 [−0.03; 0.48]	0.560 0.898 0.084
Secondary Outcomes									
Depression (PHQ-9)									
Baseline 4 weeks 8 weeks 6 months	9.19 ± 4.41 8.07 ± 3.87 6.94 ± 4.28 6.99 ± 4.15	9.34 ± 4.35 7.96 ± 3.88 6.54 ± 4.05 6.67 ± 4.12	9.17 ± 4.47 8.99 ± 4.43 8.35 ± 4.26 8.29 ± 4.18	−0.23 [−0.46; −0.00] −0.33 [−0.62; −0.05] −0.31 [−0.62; −0.01]	**0.048** **0.020** **0.045**	−0.28 [−0.52; −0.04] −0.44 [−0.68; −0.21] −0.40 [−0.69; −0.12]	**0.025** **<0.001** **0.007**	−0.05 [−0.28; 0.19] −0.11 [−0.40; 0.18] −0.09 [−0.47; 0.28]	0.706 0.465 0.620
Anxiety (GAD-7)									
Baseline 4 weeks 8 weeks 6 months	8.35 ± 3.96 7.22 ± 3.73 6.35 ± 4.09 6.37 ± 3.82	9.16 ± 4.28 6.69 ± 3.61 5.85 ± 4.14 5.55 ± 3.80	8.71 ± 4.34 8.25 ± 4.07 8.18 ± 4.79 8.10 ± 4.60	−0.22 [−0.46; 0.02] −0.36 [−0.64; −0.08] −0.37 [−0.65; −0.08]	0.077 **0.014** **0.012**	−0.46 [−0.70; −0.23] −0.58 [−0.83; −0.33] −0.66 [−0.96; −0.36]	**<0.001** **<0.001** **<0.001**	−0.23 [−0.49; 0.03] −0.20 [−0.48; 0.09] −0.28 [−0.53; −0.03]	0.082 0.166 **0.026**
Stress (PSS-4)									
Baseline 4 weeks 8 weeks 6 months	7.71 ± 2.92 6.52 ± 2.84 6.27 ± 3.16 6.02 ± 3.22	7.82 ± 3.14 6.21 ± 2.78 5.40 ± 2.81 5.29 ± 3.12	7.76 ± 3.01 7.55 ± 2.85 7.27 ± 3.14 6.79 ± 3.13	−0.35 [−0.60; −0.09] −0.31 [−0.59; −0.03] −0.23 [−0.51; 0.05]	**0.008** **0.030** 0.102	−0.47 [−0.73; −0.22] −0.60 [−0.85; −0.35] −0.47 [−0.79; −0.16]	**<0.001** **<0.001** **0.004**	−0.12 [−0.41; 0.16] −0.29 [−0.56; −0.03] −0.24 [−0.58; 0.09]	0.390 **0.031** 0.155
Well-being (WHO-5)									
Baseline 4 weeks 8 weeks 6 months	9.64 ± 4.09 11.04 ± 4.57 12.39 ± 5.13 13.19 ± 4.85	9.90 ± 4.45 12.65 ± 4.21 13.05 ± 5.13 12.94 ± 4.75	9.82 ± 4.36 10.22 ± 4.68 10.41 ± 4.79 11.22 ± 5.05	0.20 [−0.07; 0.46] 0.40 [0.13; 0.67] 0.42 [0.09; 0.75]	0.140 **0.004** **0.015**	0.52 [0.27; 0.77] 0.51 [0.24; 0.77] 0.34 [0.06; 0.61]	**<0.001** **<0.001** **0.016**	0.32 [0.05; 0.60] 0.10 [−0.21; 0.42] −0.08 [−0.37; 0.21]	**0.023** 0.508 0.589
Presenteeism (SPS)									
Baseline 4 weeks 8 weeks 6 months	54.73 ± 6.78 - 54.49 ± 7.85 54.81 ± 7.42	55.00 ± 6.60 - 55.44 ± 7.62 54.09 ± 8.12	55.41 ± 7.36 - 55.20 ± 7.66 55.29 ± 7.84	- −0.07 [−0.39; 0.25] −0.04 [−0.33; 0.24]	- 0.682 0.774	- 0.05 [−0.24; 0.33] −0.14 [−0.50; 0.22]	- 0.744 0.436	- 0.11 [−0.19; 0.42] −0.10 [−0.47; 0.27)	- 0.462 0.588
Work Output									
Baseline 4 weeks 8 weeks 6 months	63.46 ± 21.25 - 67.60 ± 23.64 69.58 ± 25.07	62.98 ± 21.65 - 71.79 ± 22.87 70.56 ± 24.11	63.09 ± 22.98 - 66.14 ± 22.61 68.94 ± 23.25	- 0.06 [−0.26; 0.37] 0.02 [−0.27; 0.31]	- 0.724 0.896	- 0.25 [−0.01; 0.50] 0.07 [−0.21; 0.35]	- 0.060 0.617	- 0.19 [−0.13; 0.51] 0.05 [−0.29; 0.39]	- 0.243 0.768
Absenteeism									
Baseline 4 weeks 8 weeks 6 months	5.95 ± 14.33 - 6.81 ± 16.63 6.14 ± 16.27	5.46 ± 8.56 - 5.40 ± 13.01 3.59 ± 7.69	6.31 ± 11.99 - 6.02 ± 17.56 4.52 ± 10.79	- 0.07 [−0.21; 0.34] 0.15 [−0.14; 0.44]	- 0.631 0.305	- −0.01 [−0.27; 0.26] −0.06 [−0.27; 0.15]	- −0.962 0.588	- −0.07 [−0.35; 0.20] −0.19 [−0.46; 0.09]	- 0.597 0.181
Interoceptive Sensibility (BPQ)									
Baseline 4 weeks 8 weeks 6 months	65.44 ± 18.68 - 66.81 ± 22.73 62.37 ± 22.46	60.20 ± 14.55 - 60.00 ± 19.10 61.17 ± 21.37	64.74 ± 18.16 - 60.89 ± 20.13 60.75 ± 19.06	- 0.26 [0.01; 0.51] 0.05 [−0.19; 0.30]	- **0.039** 0.666	- 0.09 [−0.14; 0.33] 0.17 [−0.09; 0.43]	- 0.439 0.203	- −0.17 [−0.43; 0.09] 0.12 [−0.18; 0.43]	- 0.191 0.424
Self-efficacy (SES)									
Baseline 4 weeks 8 weeks 6 months	26.40 ± 4,00 27.98 ± 3.82 28.81 ± 4.48 29.18 ± 4.90	25.67 ± 4.99 27.92 ± 4.17 29.16 ± 4.90 28.99 ± 5.09	25.74 ± 4.43 25.65 ± 4.30 25.79 ± 4.82 26.21 ± 5.15	0.45 [0.24; 0.66] 0.51 [0.29; 0.73] 0.48 [0.19; 0.76]	**<0.001** **<0.001** **0.002**	0.55 [0.33; 0.76] 0.69 [0.46; 0.92] 0.54 [0.23; 0.85]	**<0.001** **<0.001** **0.001**	0.09 [−0.14; 0.31] 0.17 [−0.07; 0.40] 0.05 [−0.27; 0.38]	0.441 0.156 0.740
Cognitive Fusion (CFQ-D)									
Baseline 4 weeks 8 weeks 6 months	31.67 ± 8.28 28.71 ± 8.24 26.36 ± 8.44 24.69 ± 8.54	32.66 ± 7.29 27.89 ± 8.37 26.13 ± 8.43 24.38 ± 8.33	31.63 ± 8.20 30.33 ± 8.50 30.58 ± 8.62 29.24 ± 9.34	−0.20 [−0.42; 0.03] −0.49 [−0.70; −0.28] −0.51 [−0.76; −0.26]	0.083 **<0.001** **<0.001**	−0.38 [−0.59; −0.17] −0.60 [−0.81; −0.38] −0.62 [−0.86; −0.39]	**<0.001** **<0.001** **<0.001**	−0.18 [−0.40; 0.04] −0.10 [−0.32; 0.12] −0.11 [−0.39; 0.17]	0.108 0.376 0.443
Alexithymia (TAS-20)									
Baseline 4 weeks 8 weeks 6 months	49.98 ± 11.20 47.33 ± 10.58 45.70 ± 10.63 44.73 ± 11.47	50.67 ± 11.83 46.86 ± 10.22 44.45 ± 10.34 43.77 ± 11.25	48.50 ± 11.67 48.16 ± 11.29 47.77 ± 11.30 47.05 ± 11.58	−0.18 [−0.36; −0.01] −0.30 [−0.47; −0.12] −0.30 [−0.52; −0.08]	**0.043** **0.001** **0.009**	−0.27 [−0.44; −0.09] −0.45 [−0.63; −0.27] −0.42 [−0.64; −0.20]	**0.003** **<0.001** **<0.001**	−0.09 [−0.28; 0.11] −0.16 [−0.34; 0.03] −0.13 [−0.36; 0.11]	0.368 0.094 0.280
Emotion Regulation–Expressive Suppression (ERQ–SP)									
Baseline 4 weeks 8 weeks 6 months	14.95 ± 5.24 14.88 ± 4.76 14.07 ± 5.19 13.65 ± 5.13	14.26 ± 4.94 13.92 ± 4.59 13.25 ± 4.65 13.40 ± 4.79	14.53 ± 5.34 15.04 5.23 14.94 ± 5.58 14.96 ± 5.52	−0.08 [−0.31; 0.14] −0.22 [−0.45; 0.01] −0.30 [−0.53; −0.07]	0.468 0.059 **0.012**	−0.19 [−0.41; 0.026] −0.29 [−0.52; −0.09] −0.27 [−0.53; −0.01]	0.083 **0.015** **0.044**	−0.12 [−0.33; 0.10] −0.08 [−0.32; 0.15] 0.02 [−0.27; 0.31]	0.289 0.481 0.894
Emotion Regulation–Cognitive Reappraisal (ERQ–RE)									
Baseline 4 weeks 8 weeks 6 months	23.98 ± 6.05 25.73 ± 5.67 27.97 ± 5.86 28.19 ± 6.57	22.78 ± 7.02 25.18 ± 6.08 26.87 ± 6.34 27.51 ± 7.03	27.61 ± 6.07 23.70 ± 6.33 24.19 ± 6.82 24.65 ± 6.69	0.33 [0.10; 0.55] 0.57 [0.31; 0.84] 0.51 [0.19; 0.82]	**0.005** **<0.001** **0.002**	0.35 [0.12; 0.57] 0.50 [0.23; 0.77] 0.48 [0.15; 0.82]	**0.003** **<0.001** **0.005**	0.01 [−0.23; 0.25] −0.09 [−0.40; 0.23] −0.02 [−0.36; 0.32]	0.943 0.581 0.908

*Notes:* BPQ—Body Perception Questionnaire; CFQ-D—Cognitive Fusion Questionnaire; CI—confidence interval; ERQ-RE—Emotion Regulation Questionnaire (Cognitive Reappraisal); ERQ-SP—Emotion Regulation Questionnaire (Expressive Suppression); FMI—Freiburg Mindfulness Inventory; GAD-7—Generalized Anxiety Disorder Questionnaire; GoD—guidance on demand; *M*—mean, *n*—number; PHQ-9—Patient Health Questionnaire; PSS-4—Short Form Perceived Stress Scale; *SD*—standard deviation; SES—Self-Efficacy Scale; SPS—Stanford Presenteeism Scale, TAS-20—Toronto Alexithymia Scale; UG—unguided; WHO-5—World Health Organization Well-Being Index. Significant comparisons (*p* ≤ 0.05) are in bold.

### 3.4. Mindfulness Reliable Improvement

At t2, participants of both UG (UG: *n* = 70, 54.3%, WL: *n* = 24, 18.9%; χ^2^(1) = 34.45, *p* < 0.001) and GoD (GoD: *n* = 84, 64.6%, WL: *n* = 24, 18.9%; χ^2^(1) = 55.1, *p* < 0.001) showed reliable improvement significantly more frequently compared with participants of WL. Reliable improvement did not significantly differ between UG and GoD (UG: *n* = 70, 54.3%, GoD: *n* = 84, 64.6%; χ^2^(1) = 2.88, *p* = 0.090). At t3, the same pattern emerged for the comparison between UG and WL (UG: *n* = 66, 51.2%, WL: *n* = 26, 20.2%; χ^2^(1) = 26.18, *p* < 0.001) and GoD and WL (GoD: *n* = 88, 67.7%, WL: *n* = 26, 20.2%; χ^2^(1) = 58.03, *p* < 0.001). In addition, the percentage of reliable improvement was significantly higher in GoD compared with UG (UG: *n* = 66, 51.2%, GoD: *n* = 88, 67.7%; χ^2^(1) = 7.34, *p* = 0.007).

**Table 4:** Effect sizes (Cohen’s *d*, 95% CI), intention-to-treat.

Variable	UG vs. WL	GoD vs. WL	UG vs. GoD
Mindfulness (FMI)			
4 weeks 8 weeks 6 months	0.76 [0.51; 1.02] 1.06 [0.80; 1.32] 0.87 [0.61; 1.13]	0.75 [0.50; 1.00] 0.91 [0.66; 1.17] 1.03 [0.77; 1.29]	−0.03 [−0.28; 0.21] −0.13 [−0.37; 0.11] 0.15 [−0.09; 0.39]
Depression (PHQ-9)			
4 weeks 8 weeks 6 months	−0.22 [−0.47; 0.02] −0.33 [−0.58; −0.08] −0.31 [−0.56; −0.07]	−0.25 [−0.49; −0.00] −0.44 [−0.68; −0.19] −0.39 [−0.64; −0.14]	−0.03 [−0.27; 0.22] −0.09 [−0.33; 0.15] −0.08 [−0.32; 0.17]
Anxiety (GAD-7)			
4 weeks 8 weeks 6 months	−0.27 [−0.51; −0.02] −0.41 [−0.66; −0.16] −0.41 [−0.66; −0.16]	−0.41 [−0.65; −0.16] −0.52 [−0.77; −0.27] −0.61 [−0.86; −0.36]	−0.14 [−0.39; 0.10] −0.12 [−0.37; 0.12] −0.22 [−0.46; 0.03]
Stress (PSS-4)			
4 weeks 8 weeks 6 months	−0.36 [−0.61; −0.11] −0.32 [−0.56; −0.07] −0.24 [−0.49; 0.00]	−0.48 [−0.72; −0.23] −0.63 [−0.88; −0.37] −0.48 [−0.73; −0.23]	−0.11 [−0.36; 0.13] −0.29 [−0.54; −0.05] −0.29 [−0.47; 0.02]
Well-being (WHO-5)			
4 weeks 8 weeks 6 months	0.18; [−0.07; 0.42] 0.40 [0.15; 0.65] 0.40 [0.15; 0.65]	0.55 [0.30; 0.79] 0.53 [0.28; 0.78] 0.35 [0.10; 0.60]	0.37 [0.12; 0.61] 0.13 [−0.12; 0.37] −0.05 [−0.30; 0.19]
Presenteeism (SPS)			
4 weeks 8 weeks 6 months	- −0.09 [−0.34; 0.15] −0.06 [−0.31; 0.18]	- 0.03 [−0.21; 0.28] −0.15 [−0.40; 0.10]	- 0.12 [−0.12; 0.37] −0.09 [−0.34; 0.15]
Work Output			
4 weeks 8 weeks 6 months	- −0.06 [0.18; 0.31] 0.03 [−0.22; 0.27]	- 0.25 [0.00; 0.49] 0.07 [−0.18; 0.31]	- −0.18 [−0.42; 0.064] −0.04 [−0.28; 0.20]
Absenteeism			
4 weeks 8 weeks 6 months	- 0.05 [−0.20; 0.29] 0.12 [−0.13; 0.36]	- −0.04 [−0.29; 0.20] −0.10 [−0.35; 0.14]	- 0.10 [−0.15; 0.34] 0.21 [−0.03; 0.46]
Interoceptive Sensibility (BPQ)			
4 weeks 8 weeks 6 months	- 0.28 [0.03; 0.52] 0.08 [−0.17; 0.32]	- −0.05 [−0.29; 0.20] 0.02 [−0.22; 0.27]	- −0.33 [−0.57; −0.08] −0.05 [−0.30; 0.19]
Self-efficacy (SES)			
4 weeks 8 weeks 6 months	0.57 [0.32; 0.82] 0.65 [0.40; 0.90] 0.59 [0.34; 0.84]	0.54 [0.29; 0.78] 0.69 [0.44; 0.94] 0.54 [0.29; 0.79]	−0.02 [−0.26; 0.23] 0.07 [−0.17; 0.32] −0.04 [−0.28; 0.21]
Cognitive Fusion (CFQ-D)			
4 weeks 8 weeks 6 months	−0.19 [−0.44; 0.052] −0.49 [−0.74; −0.25] −0.51 [−0.76; −0.26]	−0.29 [−0.54; −0.04] −0.52 [−0.77; −0.27] −0.55 [−0.80; −0.30]	−0.10 [−0.34; 0.14] −0.03 [−0.27; 0.22] −0.04 [−0.28; 0.21]
Alexithymia (TAS-20)			
4 weeks 8 weeks 6 months	−0.08 [−0.32; 0.17] −0.19 [−0.43; 0.06] −0.20 [−0.45; 0.04]	−0.12 [−0.37; 0.12] −0.31 [−0.55; −0.06] −0.29 [−0.53; −0.04]	−0.05 [−0.29; 0.20] −0.12 [−0.36; 0.12] −0.08 [−0.33; 0.16]
Emotion Regulation–Expressive Suppression (ERQ–SP)			
4 weeks 8 weeks 6 months	−0.03 [−0.28; 0.21] −0.16 [−0.41; 0.08] −0.25 [−0.49; 0.00]	−0.23 [−0.47; 0.02] −0.33 [−0.58; −0.08] −0.30 [−0.55; −0.06]	−0.21 [−0.45; 0.04] −0.17 [−0.41; 0.078] −0.05 [−0.29; 0.19]
Emotion Regulation–Cognitive Reappraisal (ERQ–RE)			
4 weeks 8 weeks 6 weeks	0.34 [0.09; 0.58] 0.60 [0.34; 0.85] 0.53 [0.28; 0.78]	0.24 [−0.01; 0.48] 0.41 [0.16; 0.65] 0.42 [0.17; 0.66]	−0.09 [−0.34; 0.15] −0.18 [−0.42; 0.06] −0.10 [−0.34; 0.14]

*Notes*: BPQ—Body Perception Questionnaire; CFQ-D—Cognitive Fusion Questionnaire; ERQ-RE—Emotion Regulation Questionnaire (Cognitive Reappraisal); ERQ-SP—Emotion Regulation Questionnaire (Expressive Suppression); FMI—Freiburg Mindfulness Inventory; GAD-7—Generalized Anxiety Disorder Questionnaire; GoD—guidance on demand; PHQ-9—Patient Health Questionnaire; PSS-4—Short Form Perceived Stress Scale; SES—Self-Efficacy Scale; SPS—Stanford Presenteeism Scale, TAS-20—Toronto Alexithymia Scale; UG—unguided, WHO-5—World Health Organization Well-Being Index.

### 3.5. Per-Protocol Analyses

The results of the sub-sample of participants that completed at least 5 modules at t2 (UG: *n* = 26; GoD: *n* = 36) mostly corresponded to the total sample concerning comparisons between IGs and WL, with improvements generally being more pronounced than in non-adherent participants (see Table S1). No significant results were found for comparisons between UG and GoD.

### 3.6. Intervention Adherence and Satisfaction

Concerning intervention adherence 8 weeks after randomization (t2), 20.4% of UG participants and 27.8% of GoD participants completed 5 of 7 modules. A total of 6 months after randomization (t3), 28.1% of UG and 39.3% of GoD fulfilled this definition. While adherence was not significantly different between the two IGs at t2 (UG: *n* = 129, 20.4%, GoD: *n* = 130, 27.8%; *χ*^2^(1) = 1.92, *p* = 0.107), follow-up adherence was significantly higher in GoD at t3 (UG: *n* = 129, 28.1%, GoD: *n* = 130, 39.3%; *χ*^2^(1) = 3.56, *p* = 0.039). The average number of modules completed at t2 was *M* = 2.51 (*SD* = 2.08) in the UG and *M* = 3.07 (*SD* = 2.32) in the GoD condition. At t3, the average number of modules completed was *M* = 3.09 (*SD* = 2.80) in UG and *M* = 3.83 (*SD* = 3.04) in GoD. By t3, 7.0% of UG participants and 10.0% of GoD participants had completed all intervention modules, including the 2 booster sessions. 

The average rating for StudiCare-M on the Client Satisfaction Questionnaire (CSQ-8) was *M* = 26.19 (*SD* = 5.44) in UG (complete-case-analysis, *n*_UG_ = 64) and *M* = 26.49 (*SD* = 5.95) in GoD (complete-case-analysis, *n*_GoD_ = 70), with a theoretical and actual range of 8–32. Group did not significantly predict satisfaction (*t*(133) = 0.30, *p* = 0.763)

When pooling both IGs, 87% partially or fully agreed that the intervention was of high quality, 85% indicated partial or full agreement that they would recommend the intervention to a friend who needed similar help, and 83% partially or fully agreed that the intervention met their needs. For the additional statements of the CSQ-8, partial or full agreement was stated as follows: “I received the kind of intervention I expected” (82%), “I am satisfied by the amount of help I received from the intervention” (79%), “Overall, I am satisfied with the intervention” (85%), “I would use such an intervention again” (77%), and “The intervention helped me cope more adequately with my problems” (76%). 

### 3.7. Negative Intervention Effects

In general, reliable deterioration of the primary outcome of mindfulness was rare. At t2, it only occurred in WL (*n* = 7, 5.4%). At t3, it was observed 9 times in WL (7.1%) and 1 time in UG (1%). No case of reliable deterioration was detected in GoD. Concerning the INEP, for both t2 (complete-case-analysis; *n*_UG_ = 64, *n*_GoD_ = 71) and t3 (complete-case-analysis; *n*_UG_ = 53, *n*_GoD_ = 54), few side effects of mostly minor intensity were reported and the number of reported side effects was comparable between groups. Concerning changes experienced by participants in different areas of life that were attributed to the intervention, 12 (UG) vs. 10 (GoD) negative effects were reported between t0 and t2 and 7 (UG) vs. 8 (GoD) negative effects between t2 and t3. Items with the most reported negative effects were “Since participating in the StudiCare training, I have been suffering more from events from my past” (*n*_t2 + t3_ = 6) and “Since participating in the StudiCare training, I have been experiencing more conflicts in my partnership.” (*n*_t2 + t3_ = 5). No participant reported suicidal ideation as a side effect of the intervention. The percentage (complete-case) of participants experiencing at least one side effect was 18.7% (*n* = 12, UG) vs. 14.1% (*n* = 10, GoD) at t2 (χ^2^(1) = 0.54, *p* = 0.464) and 13.2% (*n* = 7, UG) vs. 14.8% (*n* = 8, GoD) at t3 (χ^2^(1) = 0.57, *p* = 0.811).

Negative experiences associated with content and e-coaching were reported more frequently, mostly with minor to moderate intensity (t2: *n*_UG_ = 27, *n*_GoD_= 19; t3: *n*_UG_ = 18, *n*_GoD_ = 15). The most frequently reported negative experiences were “I felt forced by the StudiCare training or the e-coach to do exercises that I really didn’t want to do at all.” (*n*_t2 + t3_ = 43) and “By participating in StudiCare training, I spend too much time in front of the computer and neglect my hobbies and social contacts.” (*n*_t2 + t3_ = 19). None of the participants reported offensive statements associated with intervention content or e-coaching.

### 3.8. Additional Exploratory Analyses

A descriptive overview of guidance and adherence-associated measures can be found in Table 5. Exploring the influence of these variables on the primary outcome (complete-case-analysis), we found the number of modules completed at t2 (*F*(1, 135) = 12.15, *p* = 0.001, *R^2^*_Adjusted_ = 0.08), the number of mindfulness practice days per week at t2 (*F*(1, 133) = 9.16, *p* = 0.003, *R^2^*_Adjusted_ = 0.06), and the average minutes per mindfulness practice day at t2 (F(1, 137) = 7.74, *p* = 0.006, *R^2^*_Adjusted_ = 0.05) to significantly predict the FMI score of both IGs at t2. This means that for every extra module completed, an increase of 0.71 points (*b* = 0.71, *t*(136) = 3.47, *p* = 0.010), for every extra practice day per week an increase of 0.74 points (*b* = 0.74, *t*(134) = 3.03, *p* = 0.003), and for every extra minute per practice day an increase of 0.05 points (*b* = 0.05, *t*(138) = 2.78, *p* = 0.006) on the FMI was predicted. However, if these 3 variables were examined in a multivariate model (*F*(1, 130) = 6.14, *p* = 0.001, *R^2^*_Adjusted_ = 0.10), only the number of modules completed at t2 remained a significant predictor (*b* = 0.60, *t*(133) = 2.817, *p* = 0.006).

Both the group assignment (*F*(1, 255) = 3.99, *p* = 0.047, *R^2^*_Adjusted_ = 0.01; *d* = 0.25, 95% CI 0.01–0.49) and SMS coach signup (*F*(1, 255) = 5.63, *p* = 0.018, R^2^_Adjusted_ = 0.02, *d* = 0.30, 95% CI 0.06–0.55) emerged as significant predictors for number of modules completed at t2 in both IGs. Specifically, being in GoD as opposed to UG predicted an increase of 0.55 modules at t2 (*b* = 0.55, *t*(256) = 2.00, *p* = 0.047), and signing up for the SMS coach predicted an increase of 0.66 modules at t2 (*b* = 0.66, *t*(256) = 2.37, *p* = 0.018). When both variables were explored in a multivariate model (*F*(2, 254) = 4.63, *p* = 0.03, *R^2^*_Adjusted_ = 0.03), only SMS-coach signup remained a significant predictor (*b* = 0.064, *t*(256) = 2.28, *p* = 0.023).

Finally, the number of e-coach contacts significantly predicted the number of modules completed at t2 in GoD (*F*(1, 128) = 7.59, *p* = 0.007, *R^2^*_Adjusted_ = 0.05). For each additional contact, an increase of 0.56 modules was predicted (*b* = 0.56, *t*(129) = 2.75, *p* = 0.007). 

**Table 5:** Descriptive overview of guidance and adherence-associated variables.

Variable		*n*	*M (SD)*
Mindfulness practice days per week *	IG1	64	3.13 (1.77)
	IG2	71	3.58 (1.70)
Average minutes per mindfulness practice day *	IG1	68	9.18 (22.17)
	IG2	71	16.41 (25.41)
Number of modules completed	IG1	129	2.52 (2.09)
	IG2	130	3.07 (2.32)
Number of e-coach contacts (all)	IG2	130	0.28 (0.98)
Number of e-coach contacts (at least one e-coach contact)	IG2	20	1.85 (1.87)
		*n*	*N* (%)
SMS-coach signup	IG1	129	49 (38.0)
	IG2	130	56 (43.1)
At least one e-Coach contact	IG2	130	20 (15.4)

*Notes. n/N*—number; *M*—mean; *SD*—standard deviation. * completers only.

## 4. Discussion

In this three-group randomized controlled trial (RCT), we evaluated the effectiveness of two different versions (unguided, UG; guidance on demand, GoD) of the internet- and mobile-based intervention (IMI) StudiCare Mindfulness (StudiCare-M) for college students compared with a waitlist control group (WL). We found that both versions of StudiCare-M significantly improved mindfulness and various other outcomes of mental health compared with WL, with effects generally remaining stable after 6 months. Additionally, we compared the two versions against each other and found effectiveness of UG and GoD to be similar overall. We also did not find any significant differences concerning intervention satisfaction or negative effects between UG and GoD groups. However, GoD participants showed a significantly higher follow-up adherence after 6 months. 

For the primary outcome of mindfulness, we found large effects (*d* = 0.94–1.07, 95% CI 0.68 to 1.33) post-intervention when comparing both UG and GoD against WL. This is considerably larger than the moderate effect of *g* = 0.40 (95% CI 0.30–0.50) identified by a recently updated meta-analysis of RCTs that evaluated mindfulness-based IMI (*N* = 97) compared with different types of control groups [17]. In a previous trial that examined a guided version of StudiCare-M [19], we also found a large effect on mindfulness (*d* = 1.37, 95% CI 1.01 to 1.73) compared with WL. We hypothesized at the time that guidance could be a reason for the increased effectiveness compared with other mindfulness-based IMIs. However, the current trial demonstrated that the unguided and minimally guided (GoD) versions of StudiCare-M still produced a large effect on mindfulness compared with WL, with no significant difference between both versions. Another potential reason for the increased effectiveness might be our choice to utilize a waitlist control group, which previous literature suggests might be associated with an overestimation of effectiveness [68]. Despite this consideration, the effects observed in this study were still considerably larger than those found compared to inactive control groups in previous research (*g* = 0.52, 95% CI 0.41 to 0.63) [17]. Finally, the reason for the large effect of StudiCare-M on mindfulness might have been the intervention itself. It was constructed using evidence-based treatment manuals, it included case examples, audio files, and numerous meditation and self-reflection exercises that encourage regular practice [23,72], and the contents were specifically developed for and with the involvement of the target group. These design measures might have positively influenced effectiveness. 

Concerning secondary outcomes of mental health, we mostly found significant small to moderate effects in both the GoD and UG version of StudiCare-M. The effects on depressive, anxiety, and stress symptoms (*d* = −0.62 to −0.31, 95% CI −0.87 to −0.07) were comparable in size to those found in previous research (*g* = −0.44 to −0.26, 95% CI −0.55 to −0.18) [17]. In addition, our results demonstrated that psychological resources such as self-efficacy, cognitive defusion, emotional insight, and emotion regulation were promoted (*g* = −0.25 to 0.69, 95% CI 0.00 to 0.94). This is an important finding, as the strengthening of positive mental health has been postulated to reduce the incidence of mental disorders [73,74,75].

The fact that we did not find substantial differences in effectiveness between the GoD and UG versions of StudiCare-M overall is in line with previous evidence comparing guided and unguided mindfulness-based IMI [17] and GoD and unguided IMI in CBT-based IMI [24,25]. These findings are compelling because full guidance has been found to be associated with effectiveness for IMI in general [20]. For example, guided cognitive–behavioral IMIs for depression were found to have larger effects (*g* = 0.60 to 1.90) compared with unguided formats (*g* = 0.30–0.70) [76]. Explanations might be found in the treatment rationale (e.g., CBT vs. ACT/MBSR), examined populations (e.g., non-clinical vs. clinical), form/dose of guidance (e.g., full vs. GoD), or outcomes. When examining the ITT results of the current trial in detail, we did find some indications of superior long-term effectiveness of GoD concerning the reliable improvement of mindfulness. This result might be explained by the significantly increased follow-up adherence in GoD participants after 6 months. Interestingly, we also found significantly larger reductions in anxiety and stress, as well as improvements in well-being at some assessment points in GoD. Previous research suggests that perceived social support mediates changes in psychological symptoms [77], and the availability of an e-coach in the GoD condition of the current study might have led to an increase in perceived social support. However, since the actual usage of GoD was low and usually lasted no longer than two contacts, this effect might have been very small. Future research should examine under what exact circumstances guidance does or does not lead to improved effectiveness. For example, there is still insufficient knowledge regarding the dose–response relationship between guidance, adherence, and effectiveness [72]. To investigate this, different levels of guidance (e.g., full guidance vs. GoD) will have to be directly compared within one trial. Concerning the current trial, the results indicate that—at least in the context of a preventive mindfulness-based IMI for college students—GoD might not be worth the additional cost and effort, since it was not associated with substantial improvements in effectiveness and adherence compared with an UG version that incorporated adherence-fostering design elements. Finally, the lack of superiority of the GoD compared with the UG version suggests that factors such as social support [77] or therapeutic alliance [78], which are important mechanisms of change in face-to-face therapy, might be less crucial to the effectiveness of mindfulness-based IMIs. Instead, other mechanisms, including factors such as self-efficacy that are related to the self-help format, might be in play [79].

Regarding long-term effectiveness of mindfulness-based IMIs, Somers-Spijkerman and colleagues [17] determined in their updated meta-analysis that only 13 out of 97 trials (13%) included follow-up assessments of 6 months or more. The results of our 6-month follow-up assessments indicate that StudiCare-M produced long-lasting effects to promote positive indicators of mental health while alleviating negative ones. This is in line with the only other existing trial investigating long-term effects (12 months) of a guided mindfulness-based IMI in a college student sample [80]. These results are promising, but will have to be confirmed by further research that includes long-term measurements.

Concerning adherence, only about 20% of UG and 28% of GoD participants fulfilled our pre-defined criterion of completing 5 out of 7 modules 8 weeks after randomization, despite the incorporation of adherence-fostering intervention design (both UG and GoD) and additional guidance on demand (GoD). This rate could be considered somewhat low compared with previous research, where adherence to mindfulness-based IMI was found to range between 36% and 92% [17]. However, effects in the current trial were nonetheless found to be larger than or comparable to the average effect sizes found in previous research [17], even after only 4 weeks. Additionally, participants’ satisfaction with StudiCare-M was high and they indicated at post-assessment that in the last 8 weeks they had practiced mindfulness regularly (on average 10–15 min/3–4 times per week). These results suggest that participants might have already been satisfied after three to four sessions and quit the intervention not because they were unhappy, but because they were satisfied with the input and improvements they attained. Previous research on face-to-face therapy indeed suggests that failure to return after an initial session can represent successful treatment [81]. Consequently, it could be prudent to re-examine our criterion of adherence. Operationalization of adherence in the context of IMIs has been rather unsystematic so far [21,23,82]. In the future, additive studies [83,84] could shed light on the subject of how much intervention (e.g., number of modules) is needed to achieve different levels of effectiveness. Target effect sizes could then be determined depending on the nature of outcomes, population, and aims (e.g., prevention and treatment), and an evidence-based definition of adherence could be established. Finally, despite the fact that the majority of participants completed less than half of the intervention modules, this trial’s results showed significant effects on most mental health outcomes. This begs an intriguing question regarding the underlying mechanisms responsible for these improvements. This question will be addressed in a follow-up publication that examines moderators and mediators of intervention effectiveness [34]. 

In contrast to effectiveness, we did find follow-up adherence to be significantly higher in GoD vs. UG (UG) 6 months after randomization (39.3% vs. 28.1%; average completed modules: 4 vs. 3). Because GoD uptake was very low (20%), this finding suggests that simply being assigned an e-coach already results in improved adherence. Additionally, the number of e-coach contacts predicted adherence and the number of completed modules predicted mindfulness at post-assessment. This is consistent with the hypothesis that guidance might enhance effectiveness via increased adherence [20]. Indeed, the results of our per protocol analyses suggest that the effects are larger in adherent participants. However, the effect of GoD on adherence was probably too small to have significant impact on effectiveness. In comparison, in our previous study evaluating a fully guided version of StudiCare-M two-thirds of participants met the adherence criterion (of four out of five modules after six weeks) and the effects were larger than in the current trial [19]. To fully understand the relationship between guidance and the adherence and effectiveness of mindfulness-based IMIs, future research will have to compare different levels of guidance within one trial.

Surprisingly, we found the subscription to the SMS coach to be a superior predictor of adherence compared with guidance (GoD vs. UG). This finding suggests that adherence can also be effectively promoted via persuasive design elements such as technical forms of guidance, e.g., chatbots [85] or reminders, which is consistent with previous research [23,86]. Future trials should investigate how different persuasive design elements can affect adherence and effectiveness of mindfulness-based IMI. 

Finally, concerning negative effects of StudiCare-M, we only found one case of reliable deterioration on the primary outcome of mindfulness in the UG condition and none in GoD. Only around 15% of participants reported negative effects of mostly minor intensity and no one reported suicidal ideation, with similar results in UG and GoD. Britton et al. [29] found meditation-related adverse effects to occur in about 40% of participants undergoing 8-week mindfulness-based cognitive therapy (MBCT). However, results are only comparable to a limited extent, since Britton et al. employed both an intervention (MBCT) and an assessment method (Meditation Experiences Interview) that focused strongly on meditation. In conclusion, our results suggest that StudiCare-M is generally safe for (unguided) implementation. However, we note that even low-threshold preventive mindfulness-based IMIs have negative effects both related to intervention content (e.g., more conflicts in partnership and more suffering related to events of the past) and the IMI setting (e.g., too much time in front of the computer). Consequently, participants should be informed about possible negative effects and how to deal with them (e.g., face-to-face help offers and emergency contacts). Additionally, research on negative effects is practically non-existent in the field of mindfulness-based IMIs and future studies should routinely assess them [28].

### Limitations 

Concerning the interpretation of this trial’s results, some limitations need to be taken into consideration: Despite our best efforts to reduce study/assessment dropout—such as email and telephone reminders and raffles—dropout rates were quite high, especially at later assessment points. To avoid biased results, we followed intention-to-treat protocol and used a well-established multiple imputation procedure.

The use of a waitlist control group can lead to over- or underestimation of treatment efficacy [87]. In an effort to counteract this, we provided alternative support information to participants of all conditions and encouraged them to seek help if needed. Usage rates (29–36%) were comparable between groups. However, to substantiate findings on effectiveness, future studies could compare StudiCare-M with active control conditions such as psychoeducation or face-to-face mindfulness interventions.

Because the trial was powered to examine the effectiveness of StudiCare-M compared with a WL on the primary outcome of mindfulness at t2, all secondary analyses were of an exploratory nature. According to post hoc power analyses, this led to insufficient power of comparisons between UG and GoD, especially in the per-protocol analyses. Consequently, existing differences between the UG and GoD versions of StudiCare-M might not have been detected.

Data were exclusively collected via online self-report assessment, which introduces potential bias such as social desirability [88]. To account for this, a cooperating trial [36] investigated the effectiveness of StudiCare-M with psychobiological markers (e.g., hair cortisol).

Finally, we did not assess mindfulness-specific side effects. Previous research has demonstrated that the practice of meditation can be accompanied by a variety of negative effects, such as executive dysfunction, derealization, or insomnia [29]. Future trials should investigate the occurrence of meditation-related side effects in the context of mindfulness-based IMI.

## 5. Conclusions

This randomized controlled trial indicates that the internet- and mobile-based intervention (IMI) StudiCare Mindfulness constitutes a low-threshold, effective, and safe way to support college students in enhancing their psychological well-being. This holds true for both an unguided version of the intervention and a version with human “guidance on demand” (GoD). However, our results also suggest that GoD might not be a cost-efficient way to enhance the effectiveness of and adherence to mindfulness-based IMIs in the target group of college students, compared with an unguided version of the same intervention. Future trials could investigate the effects of using additional technical means, such as persuasive design, to further optimize adherence.

## Figures and Tables

**Table 1:** Intervention content (see [34], slightly modified).

Module	Aims and Content	Examples of Exercises and Assignments
1. Being in the here and now	Introducing the concept of mindfulness	Reviewing most and least mindful moments of the day; “body scan” meditation; taking a mindful walk
2. Mindful body perception	Practicing awareness of body signals	Testing one’s heartbeat perception; practicing “heart meditation”; mindful eating and drinking
3. A new perspective on stress	Distancing oneself from stress-inducing thoughts	Identifying former ways of coping with stress; learning techniques to challenge automatic thoughts; “mindful perception of body posture” meditation
4. Developing beneficial thoughts	Getting to know beneficial ways of thinking	Identifying one’s “stress patterns” and developing and internalizing beneficial thoughts; “mindful breathing” meditation
5. What makes your life valuable?	Identifying one’s values and pursuing one’s goals	Writing a speech for one’s 70th birthday; setting and pursuing goals with the SMART technique; variation of “body scan” meditation
6. Being mindful towards yourself	Learning how to appreciatively accept one’s personality traits	Exercise to identify different personality traits and corresponding automatic reactions; learning to accept and appreciate all personality traits; “loving kindness” meditation
7. Training your body and senses	Exercising the ability to enjoy and getting acquainted with the practice of yoga	Mindful chocolate eating exercise; mindful yoga exercises
Booster 1 (4 weeks after completion of module 7)	Repeating module 1 to 3 and mindfulness exercises	Choosing favorite mindfulness exercises; setting goals for their implementation in the coming weeks
Booster 2 (12 weeks after completion of module 7)	Repeating modules 4 to 7 and ensuring long-term integration of mindfulness into daily life	Reviewing pursuit of goals in the last two months; identifying potential barriers and developing solutions

**Table 2:** Baseline characteristics.

	All (*N* = 386)		UG (*n* = 129)		GoD (*n* = 130)		WL (*n* = 127)	
	*N*	%	*n*	%	*n*	%	*n*	%
Sociodemographic characteristics								
Age (*M*, *SD*)	29.85	4.80	25.99	5.26	25.46	5.07		
Female gender	289	74.9	92	71.3	102	78.5	95	74.8
Single	255	66.1	85	65.9	91	70.0	79	62.6
German citizenship	310	80.3	102	79.1	103	79.2	105	82.7
Study characteristics								
Full-time student	317	82.1	103	79.8	113	86.9	101	79.5
Number of total semesters (*M*, *SD)*	9.05	5.39	9.60	5.80	8.4	4.6	9.14	5.66
Study subject								
Psychology	51	13.2	18	14.0	18	13.8	15	11.8
Medicine and health	71	18.4	20	15.5	26	20.0	25	19.7
Business and law	45	11.7	16	12.4	14	10.8	15	11.8
Educational sciences	59	15.3	19	14.7	19	14.6	21	16.5
Engineering	36	9.3	18	14.0	11	8.5	7	5.5
Linguistics and culture	45	11.7	14	10.9	16	12.3	15	11.8
Social sciences	21	5.4	7	5.4	4	3.1	10	7.9
Mathematics and other sciences	56	14.5	17	13.2	21	16.2	18	14.2
Others	2	0.5	0	0	1	0.8	1	0.8
Previous help seeking								
Psychotherapy experience	89	23.1	23	17.8	28	21.5	38	30.7
Mindfulness experience	145	37.6	52	40.3	50	38.2	43	33.8
CEQ: Treatment credibility (*M*, *SD*)	20.36	3.78	20.47	3.80	20.06	3.89	20.56	3.66
CEQ: Treatment expectancy (*M*, *SD*)	18.15	4.04	18.24	4.27	17.98	3.69	18.24	4.16

*Notes*: CEQ—Credibility/Expectancy Questionnaire; GoD—guidance on demand; *M*—mean; *N/n*—number; *SD*—standard deviation; UG—unguided.

## Data Availability

All principal investigators were given full access to the data sets. The data set is stored on password-protected servers of Ulm University with restricted access. External researchers may obtain access to the final trial dataset (from H.B.) on request depending on to-be-specified data security and data exchange regulation agreements. To ensure confidentiality, data dispersed to any investigator or researcher will exclude any identifying participant information. Anonymized results are published in peer-reviewed journals and presented at international conferences.

## Supplementary Materials

The following supporting information can be downloaded at: https://www.mdpi.com/article/10.3390/ijerph20043208/s1, Table S1: Results of regression analyses, per protocol.

## Abbreviations

ACT	Acceptance commitment therapy
CBT	Cognitive behavioral therapy
CONSORT	Consolidated Standards of Reporting Trials
GoD	Guidance on demand
ICBT	Internet-based cognitive behavioral therapy
IMI	Internet- and mobile-based intervention
ITT	Intention-to-treat
MBSR	Mindfulness-based stress reduction
RCT	Randomized controlled trial
StudiCare-M	StudiCare Mindfulness
TAU	Treatment as usual
UG	Unguided
WHO	World Health Organization
WL	Waitlist control group
