# Supplementary material for: A Three-Armed Randomized Controlled Trial to Evaluate the Effectiveness, Acceptance, and Negative Effects of StudiCare Mindfulness, an Internet- and Mobile-Based Intervention for College Students with No and “On Demand” Guidance
Source: Int J Environ Res Public Health. 2023 Feb 11;20(4):3208. doi: 10.3390/ijerph20043208 (PMC9965996; doi:10.3390/ijerph20043208)
Supplement: Supplementary file 1 [file ijerph-20-03208-s001.zip › ijerph-2164334-supplementary.pdf]

Table S1. *Results of regression analyses, per-protocol*

	M ± *SD*			Primary Analyses				Secondary Analysis	
				UG vs. WL		GoD vs. WL		GoD vs. UG	
Variable	UG (*n*=26)	GoD (*n*=36)	WL (*n*=127)	β (95% CI)	*p*	β (95% CI)	*p*	β (95% CI)	*p*
Primary Outcome									
Mindfulness (FMI)									
Baseline	30.56 ± 4.74	29.29 ± 4.91	29.71 ± 4.71						
4 weeks	35.04 ± 5.81	35.36 ± 4.46	30.75 ± 5.32	0.73 [0.40; 1.062]	**<0.001**	0.93 [0.63; 1.23]	**<0.001**	0.15 [-0.34; 0.63]	0.546
8 weeks	37.84 ± 4.63	37.59 ± 4.40	31.50 ± 5.17	1.10 [0.75; 1.46]	**<0.001**	1.17 [0.88; 1.46]	**<0.001**	0.04 [-0.47; 0.54]	0.889
6 months	36.84 ± 5.10	38.13 ± 4.91	31.89 ± 5.29	0.87[0.49; 1.25]	**<0.001**	1.15 [0.83; 1.46]	**<0.001**	0.36 [-0.12; 0.83]	0.139
Secondary Outcomes									
Depression (PHQ-9)									
Baseline	9.19 ± 4.41	9.34 ± 4.35	9.17 ± 4.47						
4 weeks	7.35 ±3.38	6.97 ± 3.02	8.99 ± 4.43	-0.46 [-0.78; -0.13]	**0.006**	-0.50 [-0.78; -0.21]	**<0.001**	-0.06 [-0.45; 0.32]	0.747
8 weeks	6.44 ± 4.31	6.06 ± 3.32	8.35 ± 4.26	-0.51 [-0.88; -0.14]	**0.008**	-0.57 [-0.88; 0.26]	**<0.001**	-0.04 [-0.47; 0.38]	0.833
6 months	6.70 ± 4.30	5.63 ± 3.67	8.29 ± 4.18	-0.43 [-0.87; 0.00]	**0.050**	-0.65 [-1.00; -0.29]	**<0.001**	-0.22 [-0.72; 0.27]	0.365
Anxiety (GAD-7)									
Baseline	8.35 ± 3.96	9.16 ± 4.28	8.71 ± 4.34						
4 weeks	7.19 ± 4.26	6.45 ± 3.48	8.25 ± 4.07	-0.26; [-0.60; 0.07]	0.123	-0.50 [-0.81; -0.18]	**0.002**	-0.23; [-0.70; 0.23]	0.320
8 weeks	6.49 ± 3.96	5.80 ± 4.02	8.18 ± 4.79	-0.37 [-0.74; 0.01]	0.054	-0.55 [-0.86; -0.25]	**<0.001**	-0.20 [-0.72; 0.32]	0.444
6 months	6.10 ± 4.10	5.30 ± 3.70	8.10 ± 4.60	-0.44 [-0.84; -0.03]	**0.034**	-0.66 [-1.01; -0.30]	**<0.001**	-0.25 [-0.74; 0.25]	0.321
Stress (PSS-4)									
Baseline	7.71 ± 2.92	7.82 ± 3,14	7.76 ± 3.01						
4 weeks	6.08 ± 3.31	5.90 ± 2.41	7.55 ± 2.85	-0.49 [-0.86; -0.11]	**0.013**	-0.62 [-0.96; -0.27]	**<0.001**	-0.10 [-0.59; 0.40]	0.697
8 weeks	5.81 ± 3.48	5.19 ± 2.19	7.27 ± 3.14	-0.44 [-0.85; -0.03]	**0.035**	-0.71 [-1.05; -0.38]	**<0.001**	-0.27 [-0.74; 0.19]	0.242
6 months	5.75 ± 3.52	5.09 ± 2.87	6.79 ± 3.13	-0.31 [-0.73; 0.11]	0.145	-0.58 [-0.95; -0.21]	**0.002**	-0.26 [-0.74; 0.22]	0.279
Well-being (WHO-5)									
Baseline	9.64 ± 4.09	9.90 ± 4.45	9.82 ± 4.36						
4 weeks	11.69 ± 5.33	13.29 ± 3.76	10.22 ± 4.67	0.35 [-0.02; 0.71]	0.060	0.66 [0.33; 0.99]	**<0.001**	0.31 [-0.10; 0.72]	0.130
8 weeks	13.06 ± 5.56	13.65 ± 4.91	10.41 ± 4.79	0.57 [0.15; 0.99]	**0.008**	0.65 [0.33; 0.97]	**<0.001**	0.07 [-0.37; 0.51]	0.747
6 months	13.34 ± 4.86	13.31 ± 4.88	11.22 ± 5.05	0.46 [0.04; 0.88]	**0.033**	0.41[0.07; 0.76]	**0.043**	-0.05 [-0.51; 0.41]	0.838
Presenteeism (SPS)									
Baseline	54.73 ± 6.78	55.00 ± 6.60	55.41 ± 7.36						
4 weeks	-	-	-	-	-	-	-	-	-
8 weeks	51.69 ± 8.50	54.72 ± 6.69	55.20 ± 7.66	-0.41 [-0.89; 0.07]	0.090	-0.05 [-0.43; 0.34]	0.807	0.38 [-0.16; 0.92]	0.161
6 months	54.10 ± 8.00	54.00 ± 8.61	55.29 ± 7.84	-0.13 [-0.60; 0.35]	0.598	-0.15 [-0.58; 0.28]	0.492	-0.02 [-0.63; 0.58]	0.939
Work Output									
Baseline	63.46 ± 21.25	62.98 ± 21.65	63.09 ± 22.98						
4 weeks	-	-	-	-	-	-	-	-	-

8 weeks	72.25 ± 26.01	75.88 ± 19.24	66.14 ± 22.61	0.25 [-0.19; 0.69]	0.267	0.47 [0.10; 0.84]	**0.014**	0.20 [-0.32; 0.72]	0.448
6 months	73.66 ± 23.92	75.16 ± 18.66	68.95 ± 23.25	0.19 [-0.26; 0.64]	0.401	0.31 [-0.07; 0.70]	0.108	0.10 [-0.48; 0.68]	0.721
Absenteeism									
Baseline	5.95 ± 14.33	5.46 ± 8.56	6.31 ± 11.99						
4 weeks	-	-	-	-	-	-	-	-	-
8 weeks	7.41 ± 20.55	1.86 ± 5.54	6.02 ± 17.56	0.029 [-0.36; 0.41]	0.883	-0.17 [-0.54; 0.21]	0.382	-0.17 [-0.51; 0.16]	0.293
6 months	9.15 ± 31.39	1.81 ± 4.05	4.52 ± 10.79	0.23 [-0.15; 0.61]	0.229	-0.22 [-0.61; 0.18]	0.273	-0.13 [-0.36; 0.11]	0.282
Interoceptive sensibility (BPQ)									
Baseline	65.44 ± 18.68	60.20 ± 14.55	64.74 ± 18.16						
4 weeks	-	-	-	-	-	-	-	-	-
8 weeks	66.06 ± 27.80	58.37 ± 15.02	60.89 ± 20.13	0.28 [-0.09; 0.65]	0.138	0.05 [-0.28; 0.37]	0.783	-0.20 [-0.66; 0.26]	0.391
6 months	59.70 ± 24.78	58.23 ± 18.50	60.75 ± 19.06	-0.01 [-0.38; 0.36]	0.963	0.05 [-0.28; 0.37]	0.762	0.10 [-0.35; 0.56]	0.658
Self-efficacy (SES)									
Baseline	26.40 ± 4.00	25.67 ± 4.99	25.74 ± 4.43						
4 weeks	28.77 ± 4.14	28.35 ± 3.80	25.65 ± 4.30	0.64 [0.33; 0.95]	**<0.001**	0.62 [0.33; 0.91]	**<0.001**	-0.05 [-0.47; 0.36]	0.797
8 weeks	29.90 ± 5.18	29.86 ± 4.08	25.79 ± 4.82	0.74 [0.42; 1.05]	**<0.001**	0.82 [0.55; 1.08]	**<0.001**	0.05 [-0.38; 0.47]	0.826
6 months	29.67 ± 5.72	29.36 ± 4.73	26.21 ± 5.15	0.58 [0.23; 0.92]	**0.001**	0.60 [0.28; 0.92]	**<0.001**	-0.00 [-0.44; 0.43]	0.982
Cognitive fusion (CFQ-D)									
Baseline	31.67 ± 8.28	32.66 ± 7.29	31.63 ± 8.20						
4 weeks	28.96 ± 8.53	25.87 ± 8.72	30.33 ± 8.50	-0.27 [-0.56; 0.01]	0.063	-0.62 [-0.89; -0.35]	**<0.001**	-0.36 [-0.79; 0.07]	0.099
8 weeks	24.40 ± 8.81	23.88 ± 7.84	30.58 ± 8.62	-0.80 [-1.10; -0.49]	**<0.001**	-0.86 [-1.12; -0.60]	**<0.001**	-0.07 [-0.51; 0.37]	0.753
6 months	25.08 ± 8.37	23.69 ± 8.68	29.24 ± 9.34	-0.54 [-0.88; -0.21]	**0.002**	-0.69 [-0.98; -0.40]	**<0.001**	-0.17 [-0.6; 0.26]	0.431
Alexithymia (TAS-20)									
Baseline	49.98 ± 11.20	50.67 ± 11.83	48.50 ± 11.67						
4 weeks	47.46 ± 12.11	44.27 ± 8.20	48.16 ± 11.29	-0.26 [-0.50; -0.01]	**0.040**	-0.48 [-0.72; -0.23]	**<0.001**	-0.26 [-0.62; 0.11]	0.162
8 weeks	44.42 ± 11.43	42.54 ± 7.20	47.77 ± 11.30	-0.48 [-0.76; -0.2]	**<0.001**	-0.61 [-0.83; -0.38]	**<0.001**	-0.15 [-0.55; 0.25]	0.461
6 months	45.04 ± 11.58	42.12 ± 9.40	47.05 ± 11.58	-0.35 [-0.68; -0.02]	**0.035**	-0.55 [-0.82; -0.28]	**<0.001**	-0.22 [-0.62; 0.18]	0.270
Emotion Regulation - Expressive Suppression (ERQ-SP)									
Baseline	14.95 ± 5.24	14.26 ± 4.94	14.53 ± 5.34						
4 weeks	14.69 ± 4.68	13.64 ± 4.10	15.04 ± 5.23	-0.12 [-0.43; 0.20]	0.464	-0.27 [-0.56; 0.03]	0.075	-0.19 [-0.61; 0.23]	0.369
8 weeks	13.82 ± 6.00	13.10 ± 4.16	14.94 ± 5.58	-0.25 [-0.58; 0.07]	0.127	-0.33 [-0.61; -0.05]	**0.020**	-0.08 [-0.49; 0.33]	0.698
6 months	14.14 ± 4.87	13.43 ± 4.29	14.96 ± 5.52	-0.20 [-0.56; 0.16]	0.266	-0.28 [-0.60; 0.04]	0.087	-0.11 [-0.61; 0.40]	0.675
Emotion regulation - Cognitive Reappraisal (ERQ-RE)									
Baseline	23.98 ± 6.05	22.78 ± 7.02	27.61 ± 6.07						
4 weeks	25.81 ± 6.09	26.54 ± 6.71	23.70 ± 6.33	0.44 [0.12; 0.76]	**0.007**	0.61 [0.31; 0.92]	**<0.001**	0.18 [-0.28; 0.64]	0.445
8 weeks	28.87 5.01	27.83 ± 6.60	24.19 ± 6.82	0.77 [0.39; 1.15]	**<0.001**	0.68 [0.37; 0.99]	**<0.001**	-0.11 [-0.59; 0.37]	0.638
6 months	27.14 ± 6.41	27.74 ± 6.27	24.65 ± 6.69	0.43 [0.01; 0.85]	**0.045**	0.58 [0.197; 0.97]	**0.003**	0.16 [-0.34; 0.66]	0.525

Note. BPQ Body Perception Questionnaire, CFQ-D Cognitive Fusion Questionnaire, CI confidence interval, ERQ-RE Emotion Regulation Questionnaire (Cognitive Reappraisal), ERQ-SP Emotion Regulation Questionnaire (Expressive Suppression), FMI Freiburg Mindfulness Inventory, GAD-7 Generalized Anxiety Disorder Questionnaire, GoD guidance on demand, *M* mean, *n* number, PHQ-9 Patient Health Questionnaire, PSS-4 Short Form Perceived Stress Scale, *SD* standard deviation, SES Self-Efficacy Scale, SPS Stanford Presenteeism Scale, TAS-20 Toronto-Alexithymia Scale, UG unguided, WHO-5 World Health Organization Well-Being Index
